# Using the similarity theory and dimensional analysis to quantify the periodic dependence of the properties of chemical elements†

**DOI:** 10.1039/d3ra00927k

**Published:** 2023-03-14

**Authors:** Vyacheslav S. Protsenko

**Affiliations:** a Ukrainian State University of Chemical Technology Gagarin Ave., 8 Dnipro 49005 Ukraine Vprotsenko7@gmail.com

## Abstract

The similarity theory and dimensional analysis were used to establish the mathematical dependences of the properties of chemical elements on their atomic numbers in the periodic system. Dimensionless quantities of the complex type (similarity criteria) were derived, which include, among other quantities, the first ionization energies and atomic radii in their structure. The values of the proposed dimensionless quantities for eighty-six chemical elements of the first six periods of the periodic system (_1_H–_86_Rn) were calculated, and the corresponding criterion dependences were analyzed. The obtained criterion dependences were expanded in Fourier series with a finite number of terms; the characteristic features of the corresponding spectral diagrams were discussed. Analysis of the dependences of the properties of chemical elements on the atomic numbers, carried out within the framework of the mathematical apparatus of the similarity theory and dimensional analysis, led to the conclusion that the fundamental chemical law, known as the periodic law, is better called the law of oscillations and formulated as follows: the dependences of the properties of chemical elements on their atomic number have the character of a superposition of a series of oscillations.

## Abstract 2

Similarity theory and dimensional analysis were used to quantify the “periodic” law. The dependences of the properties of chemical elements on their atomic numbers are superpositions of a series of oscillations.

## Graphical abstract

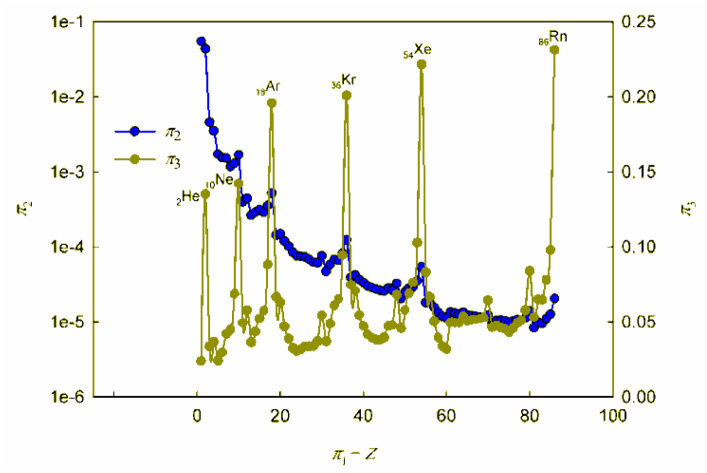

## 1. Introduction

The periodic law, which determines the regularity of changes in the physical and chemical properties of elements with an increase in the nuclear charge number, is the cornerstone of chemical science.^1–5^ Although the periodic system of elements developed by Mendeleev^1,2^ is one of the most famous achievements of modern chemistry and one of the most productive tools for chemical research, it should be recognized that the problem of establishing a strict quantitative formulation of the periodic law has not yet received a proper unambiguous solution.^6^

The problem of a quantitative description of the periodic law has been the subject of consideration in numerous previous studies. Thus, in particular, a number of simple correlations between the atomic weight of chemical elements and some empirical integers inherent in each individual element were known as early as the time of Mendeleev.^7^ Quite successful attempts were also made to interpret the structure of the periodic table from the standpoint of the concept of spatial arrangements of electrons (shells) based on the mathematical apparatus of quantum mechanics.^8–10^ Approaches of this kind are undoubtedly based on a deep and serious theoretical foundation, however, as Restrepo and Pachon rightly noted,^6^ these attempts are concentrated not so much on the general mathematical nature of the periodic law as on “the quantum-mechanically mediated electronic structure of the various atoms”.

Significant efforts have been made to interpret the periodic law within such branches of applied mathematics as information theory, theory of sets (ordered sets), quantum similarity, topological studies and cluster analysis.^11–22^ Worth mentioning is the concept developed by Pettifor,^23–26^ who proposed a phenomenological chemical scale of elements that provides structural separation and arrangement of all binary compounds with a given stoichiometry on a two-dimensional structural map. Within the framework of this theory, each atom is assigned a certain number that is different from the atomic number in the periodic system and is determined empirically. The concept proposed by Pettifor was further developed in the works of other researchers.^27–29^

Thus, it can be stated that for many decades attempts have been made to propose a mathematical formulation of the periodic law. At the same time, the numerous studies of this kind and the wide variety of approaches used indicate that this problem has not yet been adequately resolved.^30–34^

It has been repeatedly noted^6^ that a serious obstacle that makes it difficult to establish the quantitative form of the periodic law is the diversity and heterogeneity of various physical and chemical properties of elements. Indeed, if the argument of the desired quantitative form of the periodic dependence, of course, should be the charge of the atomic nucleus (*i.e.* the atomic number of the element in the periodic system), then there is no such unambiguity regarding a parameter that should be the value of the desired function. It is well known that in the scientific and educational literature it is customary to discuss the periodicity of changes in the most diverse properties of chemical elements. Such properties can be the size of an atom, ionization energy, affinity energy, electronegativity, valence, highest oxidation state, *etc.* The obvious ambiguity in the choice of a single value characterizing the properties of chemical elements makes it difficult to establish the mathematical form of the periodic law.

At the same time, up-to-date science has a powerful tool that allows combining a number of different quantitative characteristics of the physical and chemical properties of elements within a relatively small number of dimensionless combinations of these quantities that have a clear and transparent physical meaning. Obviously, such a tool is the similarity theory and dimensional analysis.

Similarity theory and dimensional analysis have a developed mathematical apparatus and are widely and successfully used in various fields of science and technology.^35,36^ These genetically related methods of quantitative data analysis are based on the idea that the influence of various factors on the properties of complex systems does not manifest itself separately, but in their combination. Therefore, in fact, in a generalized analysis, it is necessary to consider not individual quantities, but their aggregates, clearly defined for each specific process and specific system.

Combining the so-called initial quantities (usually measurable and having one or another dimension), carried out according to the methods of the theory of dimension analysis, allows deriving dimensionless complexes (the so-called similarity criteria). Establishing functional dependences between similarity criteria (criterion dependences), according to the well-known Buckingham's π-theorem,^37^ is a key way to conduct a quantitative analysis of the behaviour of various systems within the framework of similarity theory and dimensional analysis. The main advantages of this approach are the reduction in the number of analysed variables, the transition from considering individual particular cases to the analysis of generalized cases, and the ability to visually and convincingly establish the mutual interactions in the system under consideration, expressed by criterion equations of a relatively simple mathematical form.^38–41^

Similarity theory and dimensional analysis are successfully used in various fields of chemistry, chemical engineering, and related fields of science.^42–46^ However, as far as we know, attempts to use the methods of similarity theory and dimensional analysis to reveal the mathematical form of the periodic law have not yet been undertaken. Therefore, the purpose of this work was to use the mathematical apparatus of the theory of similarity and dimensional analysis for a quantitative representation of the periodic dependence of the properties of chemical elements. To achieve this goal, it seemed appropriate to consider the following questions:

(1) To select the initial values characterizing the properties of chemical elements for subsequent analysis and derive specific forms of the corresponding similarity criteria.

(2) To analyse the obtained criterion dependences.

(3) To identify a specific type of mathematical dependences of the periodic law in dimensionless coordinates of the corresponding similarity criteria.

In this paper, an attempt is made to outline the main approaches to solving all these problems.

## 2. Theory and calculations

### 2.1. Selection of initial values and derivation of the corresponding dimensionless complexes (similarity criteria)

The most important initial step, which determines the success of the implementation of the methods of similarity theory and dimensional analysis, is the correct (rational) choice of initial quantities that describe the characteristics and behaviour of the system under consideration. On the one hand, in our case, one should take into account all the main parameters that reflect the periodicity (regularity) of changes in the properties of chemical elements. On the other hand, we should avoid excessive detailing and complication of the model resulting from an excessive increase in the range of the considered initial values. For subsequent analysis, it is also important that for all (or for most) chemical elements the values of the selected initial quantities are known (measured or calculated) with satisfactory accuracy. In view of the foregoing, it should be noted that the selection of a set of initial values is carried out largely based on intuitive considerations.

The following values were chosen as the initial values in this work.

Fundamental physical constants:

(1) Electron charge (elementary charge) *Q*_e_ = 1.60210 × 10^−19^ C [C = A s];

(2) Rest mass of an electron *m*_e_ = 9.1091 × 10^−31^ kg [kg];

(3) Electric constant *ε* = 8.8542 × 10^−12^ F m^−1^ [F m^−1^ = kg^−1^ m^−3^ s^4^ A^2^];

(4) Planck constant *h* = 6.6256 × 10^−34^ J s [J s = m^2^ kg s^−1^].

Parameters of chemical elements:

(5) Charge of the nucleus of the atom *Q*_N_ [C = A s];

(6) Atomic radius *r* [m];

(7) First ionization energy (first ionization potential) of the atom *I*_1_ [J = m^2^ kg s^−2^].

Commenting on the selected values, it can be noted that it seems logical and justified to choose the charge and mass of an electron as ones of the initial values, since many chemical properties of elements are due precisely to electronic interactions. The introduction of an electric constant is caused by the fact that the forces of electrical interaction play a primary role in chemical transformations. The use of the Planck constant in the analysis is due to the need to take into account quantum mechanical effects, which are fundamentally important for understanding the chemical interaction. It is also quite obvious and natural to involve the charge of the atomic nucleus in the analysis, since this quantity will determine the argument of the desired dependence.

As regards the radius of the atom and the first ionization energy, the choice of these quantities is due to a number of important circumstances. Firstly, the values of *r* and *I*_1_ have been determined with sufficient accuracy for practically all chemical elements. Secondly, it is rightly believed that the nature of their change with increasing *Q*_N_ has a clearly defined regularity (periodicity). In this regard, several important comments should be made.

Ionization energies serve as a measure of the strength of the bond between an electron and an atomic nucleus. Changes in ionization energies are known to correlate well with changes in many properties of elements and their compounds, which is often used to predict these properties from the values of ionization energies.

At first glance, together with (or instead of) the ionization energy, one could also use the value of electron affinity. However, as is known, electron affinity does not show a strict periodicity and regularity with an increase in the atomic number, and it is characterized by a number of anomalies. In addition, electron affinity can have both positive and negative sign, which is often inconvenient. On the contrary, the ionization energy for all chemical elements is positive and determined with high accuracy. In this work, we used the values of the first ionization energies of elements given in the well-known reference book.^47^

As for the radius of the atom, there is no clear unambiguity here (the atom, as a quantum mechanical object, does not have clearly defined boundaries), and therefore, various definitions of atomic radius are known and different values can be considered: atomic radii, ionic radii, metallic radii, covalent radii, van der Waals radii, *etc.*^48–51^ To perform correct calculations, one has to use the values obtained in a consistent way for all elements. In this work, as the radius of atoms of various chemical elements, we used values borrowed from a recent publication,^29^ where they were defined as half the shortest interatomic distance in the relaxed simple cubic structure of an element.

In this paper, we limited ourselves to considering eighty-six chemical elements of the periodic system (from hydrogen to radon, *i.e.*, all elements of six complete periods of the periodic system, for which the values of the atomic radii and ionization energies are known). The dependences of the atomic radius and ionization energies on the atomic number of the element are summarized in ESI (Table S1†); they have the form (ESI, Fig. S1†), repeatedly characterized in scientific and educational literature. In order to establish the structure of respective dimensionless complexes,‡‡To avoid possible confusion, let us note that in this work the term “*complex*” implies that the considered dimensionless quantity is obtained as a result of combining several other dimensional quantities, *i.e.* has a more or less *complex mathematical structure*. We are not talking about numbers with an imaginary part. we will use the procedure, which is typical of the similarity theory and dimensional analysis.^38–41^ Firstly, dimensions of all seven chosen quantities should be tabulated (Table 1).

**Table 1:** Degrees of dimensions of physical quantities chosen for analysis (*L* – length, *M* – mass, *T* – time, and *I* – current)

Quantity and its dimension		Degree			
		*L* (m)	*M* (kg)	*T* (s)	*I* (A)
*R*_1_	*Q*_e_, C = A s	0	0	1	1
*R*_2_	*m*_e_, kg	0	1	0	0
*R*_3_	*ε*, F m^−1^ = kg^−1^ m^−3^ s^4^ A^2^	−3	−1	4	2
*R*_4_	*h*, J s = m^2^ kg s^−1^	2	1	−1	0
*R*_5_	*Q*_N_, C = A s	0	0	1	1
*R*_6_	*r*, m	1	0	0	0
*R*_7_	*I*_1_, J = m^2^ kg s^−2^	2	1	−2	0

The dimensions of all seven quantities included in Table 1 can be written as follows:

1 *R*_*j*_ = *L*^*λ*_*j*_^*M*^*μ*_*j*_^*T*^*τ*_*j*_^*I*^*υ*_*j*_^

where *λ*_*j*_, *μ*_*j*_, *τ*_*j*_, and *υ*_*j*_ are the dimensionality degrees of length *L*, mass *M*, time *T*, and current *I*, respectively.

Next, we form a matrix from the degrees of dimensions summarized in Table 1 (matrix (2)).2
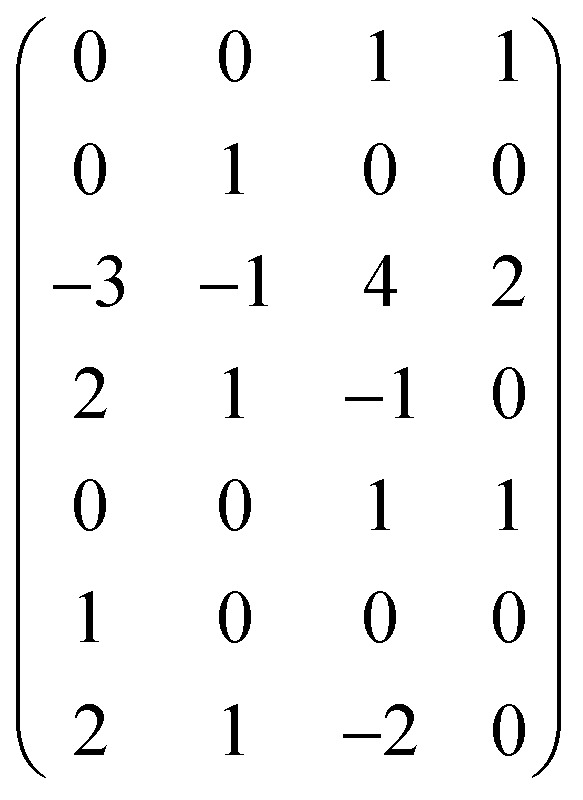


It is easy to establish that the rank of matrix (2) is equal to 4. Since the total number of dimensional quantities for this system is 7, then 7 − 4 = 3 dimensionless complex quantities (*π*_*i*_, similarity criteria) characterizing this system can be obtained:3
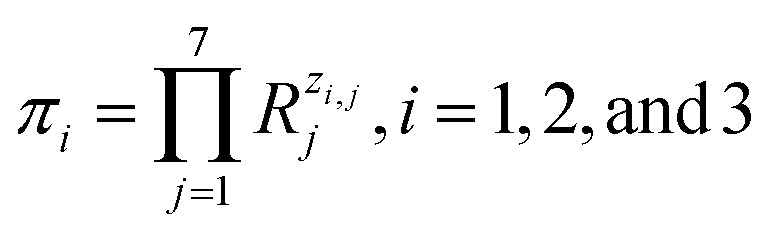


Based on eqn (1) and (3), we get the following system of four equations (for degrees of length, mass, time, and current, respectively):4
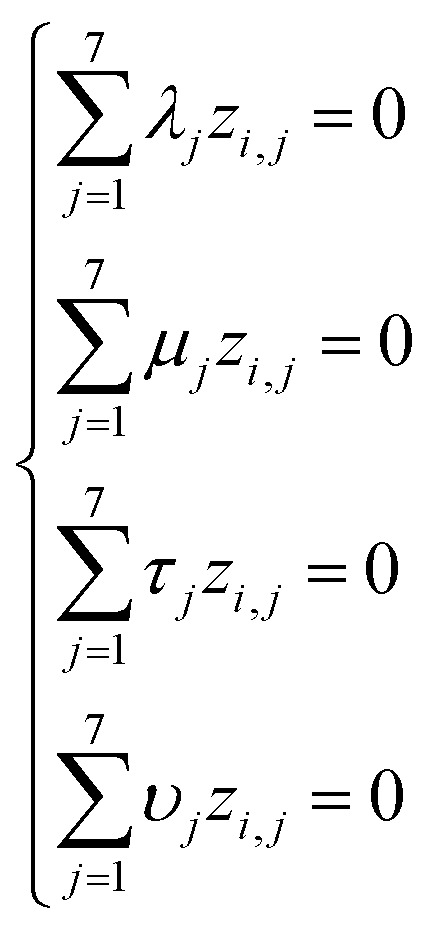
Thus, to derive dimensionless complexes, one should solve the system of linear eqn (4), which, taking into account matrix (2), has the following form:5
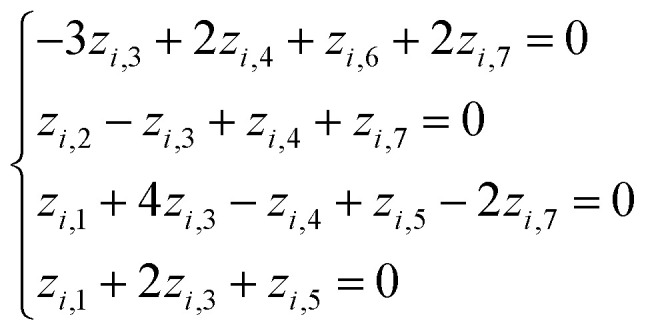


Giving the fact that the number of variables in the system of eqn (5) is 7, and the rank of matrix (2), as noted above, is 4, there are only three linearly independent solutions, *i.e.* three independent complexes *π*_*i*_. Obviously, the total number of such sets of solutions is infinitely large, and they can be determined, for example, by a fitting method. The specific form of the adopted solution is determined by considerations of the convenience of their further use and the presence of a physical meaning of certain quantities obtained. In our opinion, the most successful solutions for further analysis seem to be as follows:

First solution *z*_1,1_ = −1; *z*_1,2_ = 0; *z*_1,3_ = 0; *z*_1,4_ = 0; *z*_1,5_ = +1; *z*_1,6_ = 0; *z*_1,7_ = 0;

Second solution *z*_1,1_ = 0; *z*_1,2_ = 0; *z*_1,3_ = +1; *z*_1,4_ = 0; *z*_1,5_ = −2; *z*_1,6_ = +1; *z*_1,7_ = +1;

Third solution *z*_1,1_ = 0; *z*_1,2_ = +1; *z*_1,3_ = 0; *z*_1,4_ = −2; *z*_1,5_ = 0; *z*_1,6_ = +2; *z*_1,7_ = +1.

According to eqn (3), these three solutions correspond to the following dimensionless quantities of complex type:6
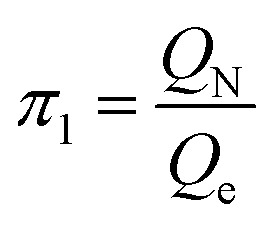
7
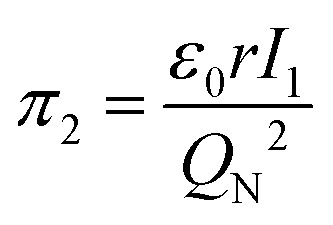
8
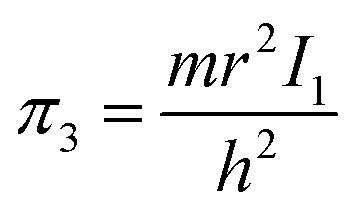


### 2.2. Physical meaning of the derived dimensionless complexes

Let us reveal the physical meaning of the obtained dimensionless complexes *π*_1_, *π*_2_ and *π*_3_.

The dimensionless complex *π*_1_ is the nuclear charge number (*i.e.* the atomic number of the chemical element in the periodic system, *Z*):

9 *π*_1_ ≡ *Z*

Obviously, this value is an argument in the desired mathematical formulation of the functional dependence, which reflects the periodic law.

To characterize the physical meaning of the dimensionless complex *π*_2_, it should be noted that in its mathematical structure, the ionization energy is compared up to a constant factor with the value of the potential energy of attraction of an electron located at a distance *r* to the nucleus given by the following expression (without taking into account the screening effect).^52^
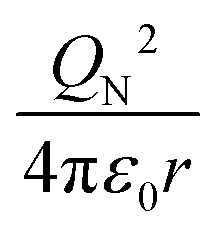


Similarly, in the mathematical structure of the dimensionless complex *π*_3_, the ionization energy, up to a constant factor, is compared with the kinetic energy of an electron located at a distance *r* from the nucleus given by the following expression.^52^
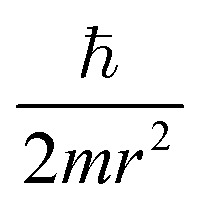


As is known,^41^ any dimensionless complex following from the analysis of dimensions (*i.e.*, any similarity criterion) is *an approximate measure of the ratio* of the relative efficiency of *two physical effects* that are essential for a given system.§§It should be emphasized that we are not talking about the exact numerical ratio of the two effects, but only about an approximate quantitative *assessment of their relative manifestation*, which is meaningful only when comparing changes in the intensity of their action as a result of changes in certain properties of the system or external conditions affecting behaviour systems. Considering this circumstance, it can be argued that dimensionless complexes *π*_2_ and *π*_3_ are measures of the ratios between the energy of detachment of an electron from an atom (ionization energy) and the potential and kinetic energy of an electron in an atom, respectively.

Such a conclusion finds indirect confirmation if we apply the reduction procedure to the Schrödinger equation, which describes the behaviour of any physical object in quantum mechanics. Let's write the Schrödinger equation for one of the simplest cases, the stationary state of an electron in a hydrogen-like atom, in the following form:

10 *Ĥψ* = *Eψ*

where the Hamiltonian *Ĥ* is defined as11
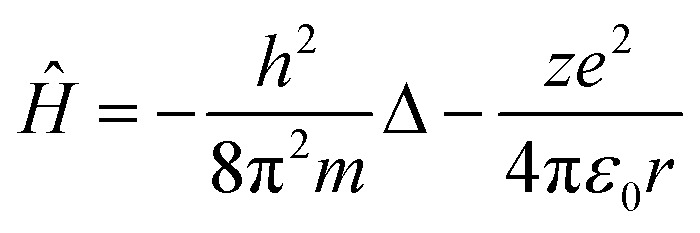


The first and second terms on the right side in eqn (11) are the kinetic and potential energy operators, respectively.

To derive dimensionless complexes from eqn (10), taking into account eqn (11), we will use the reduction procedure. Recall that the procedure of reduction of differential equations is another way to derive the mathematical structure of dimensionless complexes, alternative to the algorithm used above when analysing the dimensions of the initial physical quantities. The essence of the reduction operation lies in the fact that in the differential equation being analysed one should omit the signs of differentiation, and then divide each term of the resulting expression into any one term, arbitrarily chosen from them.^41^

Simple transformations of the Schrödinger equation according to the algorithm described above lead to two dimensionless complexes of the following form:12a
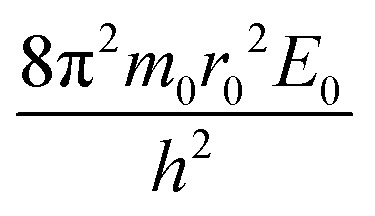
12b
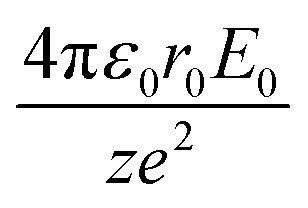
where *m*_0_, *r*_0_ and *E*_0_ are some characteristic values of mass, size and energy, which, as is customary in the similarity theory, are specified according to the conditions of a particular problem or are selected from a number of characteristic properties of the system under consideration.¶¶When choosing a particular value, certain arbitrariness is allowed, while the choice is based on heuristic considerations dictated by the convenience and expediency of using certain parameters in further analysis.

In the light of the foregoing, it is clear that in this case it is logical in eqn (12a) and (12b) to take the electron mass, the nucleus size, and the first ionization energy as characteristic quantities *m*_0_, *r*_0_ and *E*_0_, respectively.

It is easy to see that the dimensionless complexes (12) derived as a result of the reduction of the Schrödinger differential equation, up to a constant factor, coincide with the dimensionless complexes (7) and (8) obtained by analysis of dimensions. Such a coincidence, obviously, is not accidental, and is a consequence of the fact that, as the theory of similarity teaches,^41^ any equation, even very complex in its structure (including fundamental expressions like the Schrödinger equation), as a rule, expresses relatively simple ideas. Commonly, the complication of the structure of such equations results from the use in their mathematical structure of numerous directly measured initial quantities that have dimensions. It is the transition to dimensionless complexes (*i.e.* combinations of dimensional initial quantities) that makes it possible to simplify the mathematical content of the problem and clarify the physical essence of phenomena. Such a transition comes at the price of giving up the high degree of certainty that is usually achieved by applying the initial dimensional values. However, as a result, the number of considered quantitative parameters decreases, the depth and clarity of the presentation of physical relationships increases, and the analysis becomes generalized.

An interesting feature of the analysis of system behavior in the language of similarity theory and dimensional analysis is the fact that any mathematical combination of dimensionless complex quantities (similarity criteria) is also a similarity criterion. So, for example, by mathematically combining *π*_1_, *π*_2_ and *π*_3_, we can propose the following additional dimensionless quantities of complex type:13
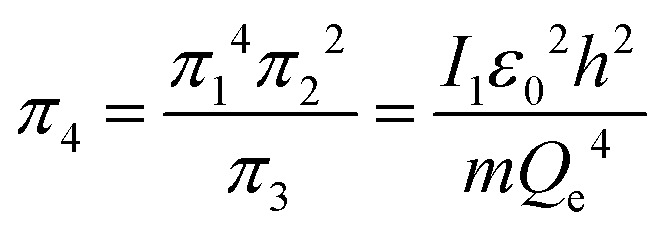
14
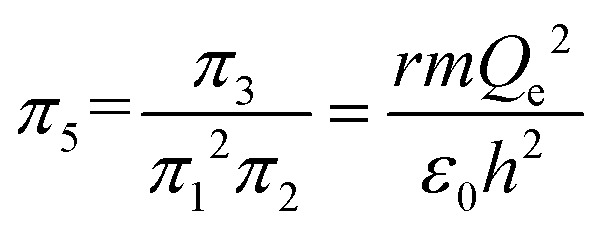


The physical meaning of the values of the complex type *π*_4_ and *π*_5_ is easy to reveal if we remember that the expression for the value of the electron energy at the 1st level of the hydrogen atom is
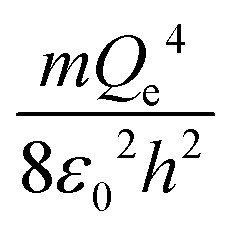
and the radius of the first Bohr orbit is:^52^
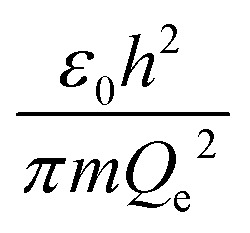


Obviously, both of these quantities are characteristic constants. It is easy to see that in the structure of the quantity of complex type (13) the first ionization energy of an atom is compared with the energy of an electron at the 1st level of a hydrogen atom (up to a constant factor). Similarly, quantity (14) allows comparing the radius of an atom with the radius of the first Bohr orbit (up to a constant factor). Thus, it becomes clear that the complex quantities *π*_4_ and *π*_5_ are expressions of the *dimensionless first ionization energy* and the *dimensionless size of the atom*, respectively.

Two important facts should be noted here. First, the structure of any dimensionless quantities of complex type, obtained *via* mathematical transformations and combinations of other dimensionless complexes, in any case will correspond to one of the possible solutions of the system of eqn (5).

Second, although system (5) in principle has an infinite number of sets of solutions, the number of simultaneously existing *linearly independent solutions*, as shown above, is *three*. Thus, the fact that new dimensionless complexes *π*_4_ and *π*_5_ were obtained as a result of the described mathematical transformation of *π*_1_, *π*_2_ and *π*_3_ does not mean that the number of analyzed quantities has increased from three to five. Strictly speaking, the quantities *π*_4_ and *π*_5_ must be analyzed *not together with*, but *instead of* the quantities *π*_2_ and *π*_3_. From the point of view of the similarity theory and dimensional analysis, such replacements do not introduce anything fundamentally new into the mathematical model of the considered system (process). The choice of a specific mathematical form and structure of dimensionless complex quantities used for analysis is determined solely by considerations of the convenience of their analysis.

### 2.3. Relationships between derived dimensionless quantities of complex type and their analysis

**Fig. 1:**
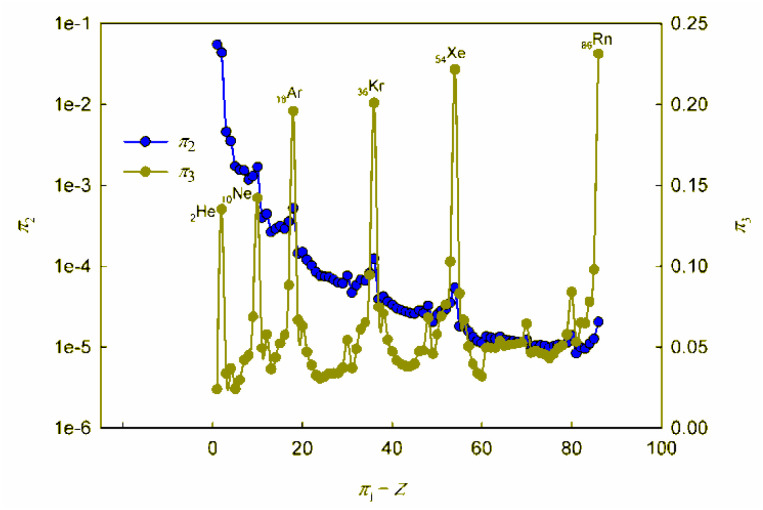
Calculated dependences *π*_2_*vs. π*_1_ and *π*_3_*vs. π*_1_. For convenience, the values on the *π*_2_-axis are plotted in logarithmic coordinates.

**Fig. 2:**
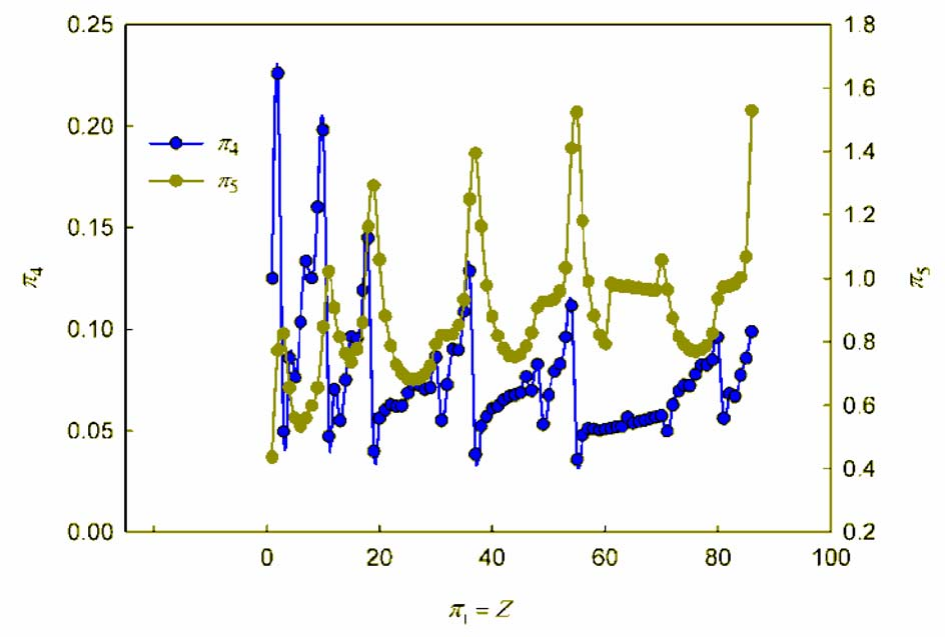
Calculated dependences *π*_4_*vs. π*_1_ and *π*_5_*vs. π*_1_.

It is obvious that in order to establish the relationship between the atomic number of an element and its physical and chemical properties within the framework of the similarity theory and dimensional analysis, it is necessary to analyze the criterion dependences *π*_2_*vs. π*_1_ and *π*_3_*vs. π*_1_. It may also be useful to consider criterion dependences in coordinates *π*_4_*vs. π*_1_ and *π*_5_*vs. π*_1_. The corresponding calculated values of these dimensionless quantities, together with the literature data on atomic radii and first ionization energies used for their calculation, are given in ESI (Table S1†). The results of calculations in the coordinates of the above criterion dependences are shown in Fig. 1 and 2.

As can be seen, these graphs reveal characteristic recurring extrema, which are also typical of the dependences of various individual properties of elements on their atomic number (see, for example, ESI, Fig. S1†). However, the graphs in Fig. 1 and 2 reflect the change in various properties not separately, but in their inextricable interaction, *i.e.*, as noted above, such an analysis acquires a theoretically justified generalized character.

The dependence of the quantity *π*_2_, which expresses a quantitative measure of the relative ratio between the energy of detachment of an electron from an atom (ionization energy) and the potential energy of an electron, has a clearly pronounced decreasing character. In addition, it is noteworthy that, with the exception of chemical elements of the first and second periods with relatively small atomic numbers, the value of quantity *π*_2_ takes on very small values (*π*_2_ ≪ 1). Because of this circumstance, the dependence *π*_2_*vs. π*_1_ is shown in Fig. 1 in semilogarithmic coordinates. If, however, this plot is presented in “normal” (not logarithmic) coordinates (ESI, Fig. S2†), it turns out that the characteristic “ripples” on this curve are practically leveled, which in fact means a rapid “degeneration” of the “periodicity” of changes in properties with increasing atomic numbers.

It is easy to understand that the numerical value of the dimensionless complex *π*_2_ allows estimating the “ease” of detachment of an electron (in energy units) in comparison with the strength of its attraction to the nucleus. The fact that the value *π*_2_ sharply decreases with increasing *Z* (*i.e.* “degenerates” from the point of view of the similarity theory||||Within the framework of the similarity theory, as already noted, any similarity criterion is a measure of the relative intensity of two effects that are important for the system. If the compared effects are incommensurable in scale, then the corresponding criterion takes on a value much greater/less than unity, thus degenerating and losing its meaning for quantitative analysis.^38–41^) means that for most chemical elements, except for the lightest ones, the first ionization energy is much less than the potential energy of attraction of the electron to the nucleus (without taking into account the screening effect). From the viewpoint of the chemical interaction, the relative “facilitation” of the detachment of an electron from an atom indicates an increase in the metallic properties.

Thus, the detected trend of a sharp decrease in the value of the similarity criterion *π*_2_ with an increase in the atomic number of a chemical element in the periodic system and its rapid degeneration reflect one of the main properties of the arrangement of elements in the periodic system: an increase in metallic properties with an increase in the charge of the atomic nucleus. Noticeable violations of this trend are found only for inert gases. It is clear that this behavior is due to the high stability of complete electron shells in atoms of inert gases.

Unlike the complex *π*_2_, which is degenerate for most chemical elements, the dimensionless complex *π*_3_ takes moderate values (not very large and not very small) for all considered elements of the first six periods (*i.e.*, this is not a degenerate similarity criterion). In this complex, the first ionization energy is compared with the kinetic energy of the electron. The latter, as follows from considering the structure of expressions (8) and (12a), is determined by the size (radius) of a given atom. From this it becomes clear that the importance and uniqueness of the quantity *π*_3_ in the analysis of the “periodic law” lies in the fact that in the mathematical structure of this parameter and, accordingly, in its numerical value, it is possible to take into account the influence of the atomic number both on the first ionization energy and on the size of the atom in their inextricable interaction. Such an approach is impossible or, at least, extremely difficult to implement with a “traditional” separate analysis of the influence of *Z* on the atom size and ionization energy.

For most of the chemical elements considered, the values of the dimensionless complex *π*_3_ fluctuate in a rather narrow range (approximately from 0.02 to 0.10). Characteristic “pulsations” (or fluctuations) in the *π*_3_*vs. π*_1_ dependence corresponding to an anomalous increase in the first ionization energy occur for inert gases and, to a much lesser extent, for halogens, as well as elements such as Be, Mg, Ca, Sr, Ba, Zn, Cd, Hg, and all lanthanides (especially Yb). The noted features are apparently due to the specifics of the filling of electronic subshells. Indeed, the outer electron shell is completely filled for atoms of inert gases, and this filling is almost completed for halogens. For alkaline earth metals, zinc, cadmium and mercury, the filling of the corresponding s-orbitals is completed, and for ytterbium, the filling of 4f^14^6s^2^-orbitals is completed.

Turning to the data analysis in Fig. 2, it should be noted that since the complex-type quantities *π*_4_ and *π*_5_ represent the dimensionless ionization energies and atomic radii, respectively, their changes with an increase in the atomic number of the chemical element and the corresponding dependences *r vs. Z* and *I*_1_*vs. Z* (ESI Fig. S1†) are symbate. Thus, in particular, the dependence of the dimensionless atomic radius in Fig. 2 clearly exhibits a sharp jump-like increase for alkali metals due to the beginning of the filling of a new outer shell, which largely determines the oscillatory nature of the curve under consideration. As for the ionization energy, as is known, the most dramatic changes that determine the oscillations in the plot of the dependence on the atomic number of the element are observed for inert gases (a very stable electronic configuration of completely filled shells, correspondingly high ionization energies) and for the following alkali metal atoms (a single electron in a new electron shell, correspondingly low ionization energies). All other features and regularities in these dependences are also well described and interpreted in the literature, so there is no need to dwell on their detailed discussion here.

Summarizing, we note that the graphs in Fig. 1 and 2, obtained in the coordinates of dimensionless quantities of a complex type in the language of similarity theory and dimensional analysis, reflect the patterns of change in the basic properties of chemical elements with increasing atomic number in a *generalized form*. In our opinion, these dependences should be considered as mathematical expressions of the “periodic” law. In this case, the question arises of representing these dependences not only in graphical forms, but also in the form of certain mathematical formulae.

Analyzing the data obtained, it should be noted that, despite the presence of certain regularities and a number of local extrema (both minima and maxima), these functions can by no means be called periodic in the classical sense of this mathematical term.**As is known, a periodic function is a function *f*(*x*) for which, for any *x*, the equality *f*(*x* + *T*) = *f*(*x*) takes place, where *T* is the period of this function. Indeed, the observed regularities of changes in the properties of chemical elements do not repeat through strictly defined intervals of change in the element's atomic number. Thus, the “periodic” law, strictly speaking, is *not a periodic function*, which has already been noted in the literature.^6,53^ Different periods in the periodic table include different numbers of chemical elements. The reason why chemists have been using terminology that is obviously distorted from the point of view of elementary mathematics for more than 150 years is the tradition that originated with Mendeleev, who first proposed the term “periodic law”. Such inaccuracy stemmed from the fact that Mendeleev did not consider the quantitative mathematical aspects of the pattern he established. This oversight in no way detracts from the significance of the brilliant conjecture of the discoverer of the “periodic law”. At the same time, respect for established traditions should not obscure the need for chemists to use correct mathematical terminology. Therefore, it seems to us that the regularity in the change in the properties of chemical elements, the presence of which is also clearly visible within the framework of the generalized mathematical apparatus developed by the similarity theory and dimensional analysis (Fig. 1 and 2), is better called *the law of oscillations*.

However, the refinement of mathematical terminology still does not solve the problem of deriving a specific type of the corresponding dependences, reflecting the mathematical law of these oscillations, and the correct formulation of this law. In our opinion, the form of the obtained criterion dependences *π*_2_*vs. π*_1_, *π*_3_*vs. π*_1_, *π*_4_*vs. π*_1_ and *π*_5_*vs. π*_1_ suggests that they can be *a superposition of several periodic functions with different periods*. The characteristic oscillatory form of these dependences allows us to assume that we can try to represent them as a linear combination of trigonometric functions, using the expansion in Fourier series with a finite number of initial terms:15
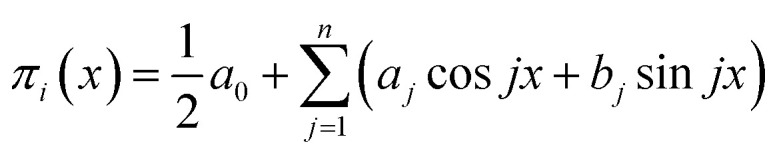
where *a*_*j*_ and *b*_*j*_ are the coefficients of the Fourier series; *x* is the argument of trigonometric functions in the series; ††††The function *π*_*i*_(*N*) in this work is approximated on a finite interval (1; 86), since the atomic numbers of chemical elements under study take values from 1 to 86. In this case, when passing to the “usual” expansion of the function *π*_*i*_(*x*) in the Fourier series, the expansion interval (0; 2π) will be divided into 85 equal segments with the abscissas of the boundaries *x* = 2π*k*/85 (*k* = 0,…, 85). Here π (without any subscript) is the numerical value of pi. and *n* is the maximum value of the terms of the Fourier series (*n* < (85/2) ≈ 42).

Series (15) can be conveniently written as a sum of simple harmonics:16
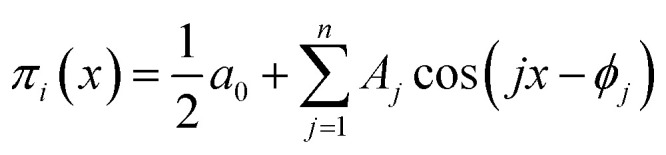


**Fig. 3:**
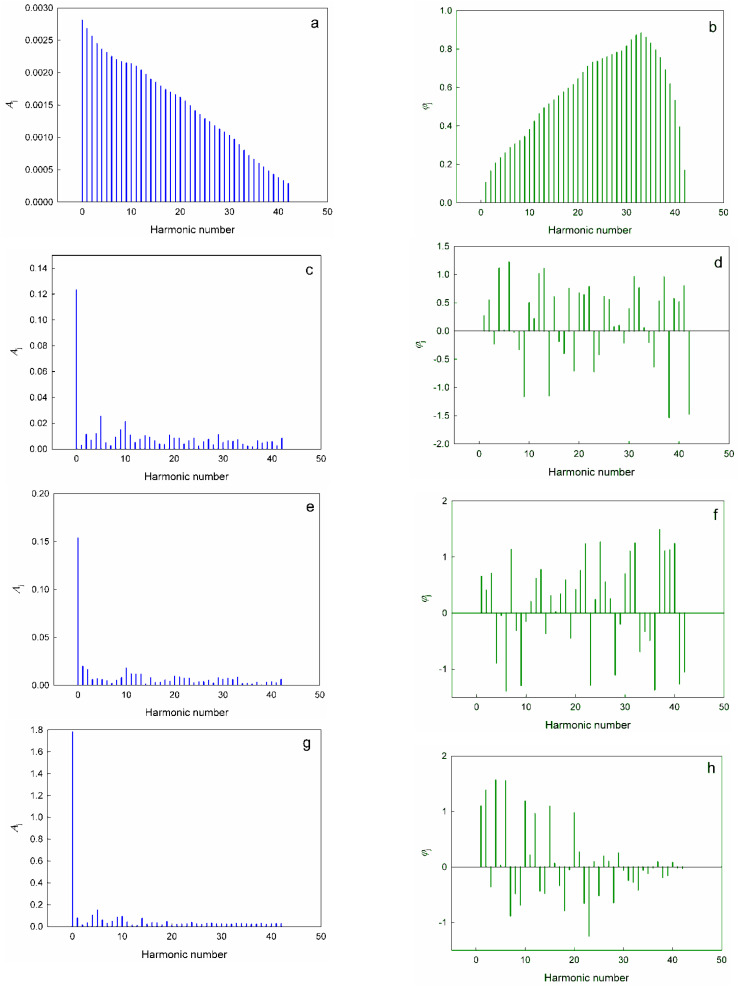
Amplitude spectra (a, c, e, and g) and frequency spectra (b, d, f, and h) of the expansions of the dimensionless complex quantities *π*_2_ (a and b), *π*_3_ (c and d), *π*_4_ (e and f) and *π*_5_ (g and h) in the Fourier series (16).

Formulae for the approximate calculation of the values of *a*_*j*_, *b*_*j*_, *A*_*j*_ and *φ*_*j*_ are given in ESI†. The calculated values of the expansion coefficients are summarized in Tables S2–S5 (ESI†). The spectra of amplitudes and frequencies for different harmonics of the expansion of the dimensionless complex quantities *π*_2_, *π*_3_, *π*_4_ and *π*_5_ in the Fourier series (16) are shown in Fig. 3.

First of all, it is noteworthy that the spectral diagrams of *π*_2_ resemble the continuous spectrum of a non-periodic process, thereby differing significantly from the spectra for other dimensionless complexes: a smooth and continuous attenuation of the amplitude of the successive harmonics is observed in Fig. 3a. Apparently, this feature is a consequence of the above-noted degenerate nature of the change of *π*_2_ with an increase in the atomic number of elements.

On the contrary, *π*_3_, *π*_4_ and *π*_5_ demonstrate typical line spectra, on which it is easy to identify a number of harmonics with increased amplitudes (*i.e.*, the most significant harmonic components of the considered oscillatory process):‡‡‡‡Specified in descending order of amplitude values.

For *π*_3_ – *j* = 0; 5; 10; 9; 4; 2; 29; 11; 19; 14;

For *π*_4_ – *j* = 0; 1; 10; 2; 11; 13; 12; 20;

For *π*_5_ – *j* = 0; 5; 4; 10; 9; 1; 14; 6.

It seems to us that the resulting expansions in Fourier series are not only a convenient formal mathematical tool that describes oscillations of the values *π*_3_, *π*_4_ and *π*_5_, but also reflect the real physical processes and phenomena behind the observed changes. Thus, if we make the transition from the expansion scale of Series (16) in the interval (0; 2*π*) to the corresponding values of the atomic numbers of chemical elements in the considered interval (1; 86), then it is easy to see that harmonics with intense amplitudes *j* = 9, 10 and 11 correspond to Δ*Z* ≈ 8, and, for example, the harmonic *j* = 5 (the second most intense amplitude in the spectral diagrams for *π*_3_ and *π*_5_) corresponds to Δ*Z* ≈ 14. In this regard, it can be noted that a change in the atomic number of elements equal to eight corresponds to the complete filling of the s and p orbitals of each electron shell, starting from the second period, and determines the number of chemical elements in the second and third periods of the periodic system. The repeated changes in the properties of successive eights of elements of the second and third periods are the most striking and visual manifestations of the “periodic” law, which served as one of the main starting points for its formulation by Mendeleev. As for the value Δ*Z* = 14, it is obvious that this corresponds to the number of chemical elements-lanthanides and corresponds to the complete filling of f-orbitals on the 6th (and subsequent) electron shell.

However, a detailed discussion of the quantitative parameters of individual terms in the expansion in the Fourier series (15), (16) and a detailed quantum-mechanical substantiation of the observed patterns are far beyond the scope of this work.

Summarizing all of the above, we can propose a refined formulation of the periodic law, which, as already noted, would be more accurate to call *the law of oscillations*:

*The dependences of the properties of chemical elements on the charge of the atomic nucleus (atomic number of the element in the periodic system) have the character of a superposition of a series of oscillations.*

## 3. Concluding remarks

Thus, in this work, we showed for the first time the possibility and fruitfulness of using the mathematical apparatus of the similarity theory and dimensional analysis to reveal the mathematical form of the “periodic” law, which is one of the basic laws of chemistry and the basis of chemical systematics. The corresponding dimensionless complexes (similarity criteria) have been derived. The physical meaning of the dimensionless complexes has been analyzed and the nature of the dependences of these dimensionless quantities on the atomic numbers for chemical elements of the first six periods of the periodic system has been discussed. It was found that the dependences obtained are oscillatory. The expansion of these dependences in a Fourier series has been carried out and analyzed.

In conclusion, it would be useful to point out some problems and outline possible directions for further research in the area concerned.

First, taking into account the flexibility of the mathematical apparatus of the applied mathematical procedures and the possibility of various modifications of the obtained dimensionless complexes (similarity criteria), it seems appropriate to search for new, convenient for analysis and visual in their meaning dimensionless quantities of a complex type that characterize the change in various physical and chemical properties of elements with an increase in the atomic number. Possibly, it may be fruitful and useful to involve some other initial dimensional quantities in addition to those used in this work.

Secondly, we should pay special attention to the correctness of the numerical values taken from the literature data and affecting the final result of the calculations. First of all, we are talking here about the radius of the atom, which, as already noted, is not a uniquely defined value, and for which, for this reason, different values are given in the literature for the same element. In this work, as mentioned above, we used the values of atomic radii borrowed from the recent work.^29^ In order to assess how much the method of determining or calculating the size of atoms of chemical elements will affect the main results formulated in this study, we additionally used values of atomic radii computed from a well-known theoretical models developed by Clementi *et al.*^51^ Using those values, alternative calculations were made according to the full algorithm above, and the results of those calculations are shown in ESI (Fig. S3–S5†). As can be seen, obviously, in this case, we get other values of the dimensionless complexes and the corresponding criterion dependences have a form slightly different from that shown above in Fig. 1 and 2. The form of the spectral diagrams (Fig. S5†) corresponding to the expansion into Fourier series also differs,§§§§Obviously, the spectral diagrams in Fig. 3e and f, and in Fig. 5Se and f, for the dimensionless complexes *π*_4_ completely coincide, since the expression of this quantity does not contain the atomic radius. although many of the most intense harmonics coincide with those observed in Fig. 3. One way or another, the discovered and quite expected differences in the numerical values of dimensionless complexes in no way affect the general conclusions formulated above (the dependences of the main properties of chemical elements, expressed in generalized coordinates of the similarity theory and dimensional analysis, are a superposition of a series of oscillations). At the same time, such ambiguity in the values of the radii of the atoms, determined experimentally or calculated theoretically by various methods, is a serious problem that makes it difficult to establish exact quantitative patterns. In the future, it remains to be established which series of values of atomic radii from those described in the literature is the most successful for quantifying the functions describing the “periodic law”, and whether the only “correct” and unambiguous choice is possible here in principle.

Thirdly, it would be expedient to involve the chemical elements of the seventh period of the periodic system in the quantitative analysis. Such an approach would make it possible to refine the derived quantitative dependences and obtain more reliable conclusions.

Fourthly, a detailed quantitative analysis of the features of oscillatory processes that are responsible for the discovered evolution of the values of dimensionless complexes that describe changes in the properties of elements in a generalized form seems fruitful. It is important to identify the most significant harmonics of the expansion of such dependences and establish those that can be discarded as insignificant for characterizing the physical meaning without loss of approximation accuracy. It is possible that instead of expansion dependences into trigonometric series, other mathematical expressions that reflect the revealed principle of superposition of oscillations in the properties of chemical elements will be more convenient for practice and more accurate.

Finally, a very important task is to establish correlations between the discovered characteristics of oscillatory functions (for example, the parameters of spectral diagrams for expansion in Fourier series) and the quantum mechanical effects behind them.

## Conflicts of interest

There are no conflicts to declare.

## Supplementary Material

RA-013-D3RA00927K-s001
