# Supplementary material for: Using the similarity theory and dimensional analysis to quantify the periodic dependence of the properties of chemical elements
Source: RSC Adv. 2023 Mar 14;13(12):8327–37. doi: 10.1039/d3ra00927k (PMC10011971; doi:10.1039/d3ra00927k)
Supplement: RA-013-D3RA00927K-s001 [file RA-013-D3RA00927K-s001.pdf]

Electronic Supplementary Material (ESI) for RSC Advances.
This journal is © The Royal Society of Chemistry 2023

Electronic Supplementary Information

Using the similarity theory and dimensional analysis to quantify the periodic dependence of the properties of chemical elements

Vyacheslav S. Protsenko

Table S1 Literature and calculation data used for analysis

Chemical element	Atomic number, $\pi_1 \equiv Z$	Atomic radius, r / Å [29]	Ionization energy, I_1 / eV [47]	Dimensionless quantities of complex type			
				$\pi_2 = \frac{\varepsilon_0 r I_1}{Q_N^2}$	$\pi_3 = \frac{m r^2 I_1}{h^2}$	$\pi_4 = \frac{I_1 \varepsilon_0^2 h^2}{m Q_e^4}$	$\pi_5 = \frac{r m Q_e^2}{\varepsilon_0 h^2}$
H	1	0.727	13.598	5.464E-02	2.389E-02	1.250E-01	4.372E-01
He	2	1.286	24.587	4.369E-02	1.352E-01	2.260E-01	7.734E-01
Li	3	1.374	5.392	4.550E-03	3.384E-02	4.956E-02	8.263E-01
Be	4	1.09	9.323	3.510E-03	3.682E-02	8.569E-02	6.555E-01
B	5	0.933	8.298	1.712E-03	2.401E-02	7.626E-02	5.611E-01
C	6	0.891	11.26	1.540E-03	2.971E-02	1.035E-01	5.358E-01
N	7	0.932	14.534	1.528E-03	4.196E-02	1.336E-01	5.605E-01
O	8	0.997	13.618	1.173E-03	4.500E-02	1.252E-01	5.996E-01
F	9	1.089	17.423	1.295E-03	6.868E-02	1.601E-01	6.549E-01
Ne	10	1.409	21.565	1.679E-03	1.423E-01	1.982E-01	8.474E-01
Na	11	1.701	5.139	3.993E-04	4.943E-02	4.723E-02	1.023E+00
Mg	12	1.508	7.646	4.426E-04	5.780E-02	7.027E-02	9.069E-01
Al	13	1.355	5.986	2.653E-04	3.653E-02	5.502E-02	8.149E-01
Si	14	1.269	8.152	2.917E-04	4.364E-02	7.492E-02	7.632E-01
P	15	1.223	10.487	3.151E-04	5.214E-02	9.638E-02	7.355E-01
S	16	1.293	10.36	2.892E-04	5.757E-02	9.522E-02	7.776E-01
Cl	17	1.431	12.968	3.549E-04	8.827E-02	1.192E-01	8.606E-01
Ar	18	1.933	15.76	5.197E-04	1.957E-01	1.448E-01	1.162E+00
K	19	2.151	4.341	1.430E-04	6.676E-02	3.990E-02	1.294E+00
Ca	20	1.761	6.113	1.488E-04	6.301E-02	5.618E-02	1.059E+00
Sc	21	1.466	6.562	1.206E-04	4.688E-02	6.031E-02	8.816E-01
Ti	22	1.308	6.828	1.020E-04	3.883E-02	6.275E-02	7.866E-01
V	23	1.209	6.746	8.522E-05	3.278E-02	6.200E-02	7.271E-01
Cr	24	1.162	6.767	7.545E-05	3.037E-02	6.219E-02	6.988E-01
Mn	25	1.136	7.434	7.468E-05	3.189E-02	6.832E-02	6.832E-01
Fe	26	1.131	7.902	7.307E-05	3.360E-02	7.263E-02	6.802E-01
Co	27	1.137	7.881	6.794E-05	3.387E-02	7.243E-02	6.838E-01
Ni	28	1.16	7.64	6.248E-05	3.417E-02	7.022E-02	6.976E-01
Cu	29	1.203	7.726	6.108E-05	3.717E-02	7.101E-02	7.235E-01
Zn	30	1.32	9.394	7.615E-05	5.441E-02	8.634E-02	7.938E-01
Ga	31	1.365	5.999	4.710E-05	3.715E-02	5.514E-02	8.209E-01
Ge	32	1.365	7.899	5.820E-05	4.892E-02	7.260E-02	8.209E-01
As	33	1.369	9.789	6.802E-05	6.098E-02	8.997E-02	8.233E-01
Se	34	1.418	9.752	6.612E-05	6.518E-02	8.963E-02	8.528E-01
Br	35	1.551	11.814	8.268E-05	9.447E-02	1.086E-01	9.328E-01
Kr	36	2.077	14	1.240E-04	2.008E-01	1.287E-01	1.249E+00
Rb	37	2.319	4.177	3.911E-05	7.467E-02	3.839E-02	1.395E+00
Sr	38	1.935	5.695	4.218E-05	7.088E-02	5.234E-02	1.164E+00
Y	39	1.625	6.217	3.671E-05	5.457E-02	5.714E-02	9.773E-01
Zr	40	1.463	6.634	3.353E-05	4.720E-02	6.097E-02	8.798E-01
Nb	41	1.362	6.759	3.027E-05	4.168E-02	6.212E-02	8.191E-01
Mo	42	1.294	7.092	2.875E-05	3.947E-02	6.518E-02	7.782E-01
Tc	43	1.257	7.28	2.735E-05	3.824E-02	6.691E-02	7.559E-01
Ru	44	1.249	7.361	2.625E-05	3.817E-02	6.765E-02	7.511E-01
Rh	45	1.264	7.459	2.573E-05	3.961E-02	6.855E-02	7.602E-01

Pd	46	1.306	8.337	2.844E-05	4.727E-02	7.662E-02	7.854E-01
Ag	47	1.379	7.576	2.614E-05	4.789E-02	6.963E-02	8.293E-01
Cd	48	1.509	8.994	3.256E-05	6.808E-02	8.266E-02	9.075E-01
In	49	1.541	5.786	2.053E-05	4.567E-02	5.318E-02	9.267E-01
Sn	50	1.541	7.344	2.502E-05	5.797E-02	6.750E-02	9.267E-01
Sb	51	1.553	8.608	2.841E-05	6.901E-02	7.911E-02	9.340E-01
Te	52	1.596	9.01	2.939E-05	7.629E-02	8.281E-02	9.598E-01
I	53	1.721	10.451	3.539E-05	1.029E-01	9.605E-02	1.035E+00
Xe	54	2.344	12.13	5.389E-05	2.215E-01	1.115E-01	1.410E+00
Cs	55	2.535	3.894	1.804E-05	8.318E-02	3.579E-02	1.525E+00
Ba	56	1.962	5.212	1.802E-05	6.669E-02	4.790E-02	1.180E+00
La	57	1.647	5.577	1.563E-05	5.029E-02	5.126E-02	9.905E-01
Ce	58	1.467	5.539	1.335E-05	3.962E-02	5.091E-02	8.822E-01
Pr	59	1.367	5.473	1.188E-05	3.400E-02	5.030E-02	8.221E-01
Nd	60	1.32	5.525	1.120E-05	3.200E-02	5.078E-02	7.938E-01
Pm	61	1.635	5.582	1.356E-05	4.960E-02	5.130E-02	9.833E-01
Sm	62	1.626	5.644	1.320E-05	4.960E-02	5.187E-02	9.779E-01
Eu	63	1.62	5.67	1.279E-05	4.946E-02	5.211E-02	9.743E-01
Gd	64	1.623	6.15	1.347E-05	5.385E-02	5.652E-02	9.761E-01
Tb	65	1.613	5.864	1.237E-05	5.071E-02	5.389E-02	9.700E-01
Dy	66	1.613	5.939	1.216E-05	5.136E-02	5.458E-02	9.700E-01
Ho	67	1.604	6.022	1.189E-05	5.150E-02	5.535E-02	9.646E-01
Er	68	1.602	6.108	1.170E-05	5.211E-02	5.614E-02	9.634E-01
Tm	69	1.602	6.184	1.150E-05	5.275E-02	5.684E-02	9.634E-01
Yb	70	1.759	6.254	1.241E-05	6.432E-02	5.748E-02	1.058E+00
Lu	71	1.605	5.426	9.549E-06	4.646E-02	4.987E-02	9.652E-01
Hf	72	1.454	6.825	1.058E-05	4.796E-02	6.273E-02	8.744E-01
Ta	73	1.358	7.55	1.063E-05	4.628E-02	6.939E-02	8.167E-01
W	74	1.316	7.864	1.045E-05	4.527E-02	7.228E-02	7.914E-01
Re	75	1.287	7.834	9.907E-06	4.313E-02	7.200E-02	7.740E-01
Os	76	1.278	8.438	1.032E-05	4.581E-02	7.755E-02	7.686E-01
Ir	77	1.288	8.967	1.077E-05	4.945E-02	8.241E-02	7.746E-01
Pt	78	1.311	8.959	1.067E-05	5.118E-02	8.234E-02	7.884E-01
Au	79	1.374	9.226	1.123E-05	5.790E-02	8.479E-02	8.263E-01
Hg	80	1.556	10.438	1.403E-05	8.400E-02	9.593E-02	9.358E-01
Tl	81	1.617	6.108	8.320E-06	5.309E-02	5.614E-02	9.724E-01
Pb	82	1.622	7.417	9.889E-06	6.486E-02	6.817E-02	9.755E-01
Bi	83	1.635	7.286	9.558E-06	6.474E-02	6.696E-02	9.833E-01
Po	84	1.67	8.417	1.101E-05	7.803E-02	7.736E-02	1.004E+00
At	85	1.777	9.318	1.267E-05	9.780E-02	8.564E-02	1.069E+00
Rn	86	2.544	10.749	2.044E-05	2.312E-01	9.879E-02	1.530E+00

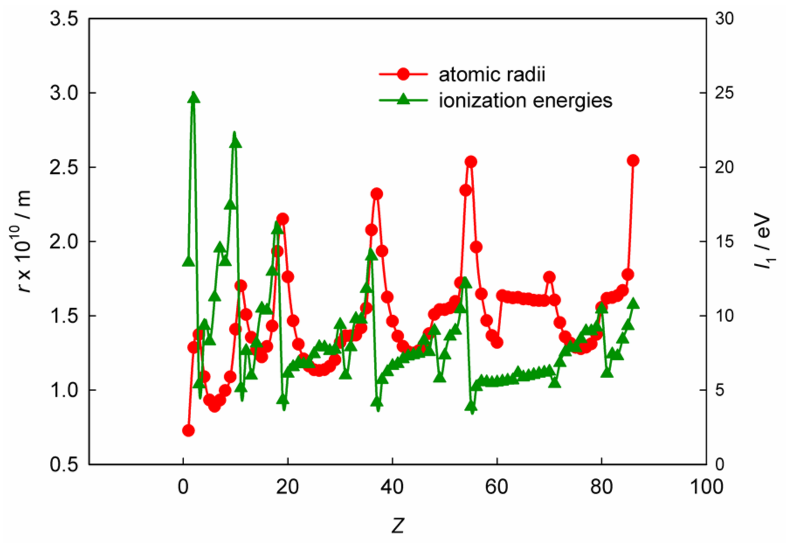

Fig. S1 Dependences of the radius of atoms and the first ionization energy on the atomic number of the elements in the periodic system.

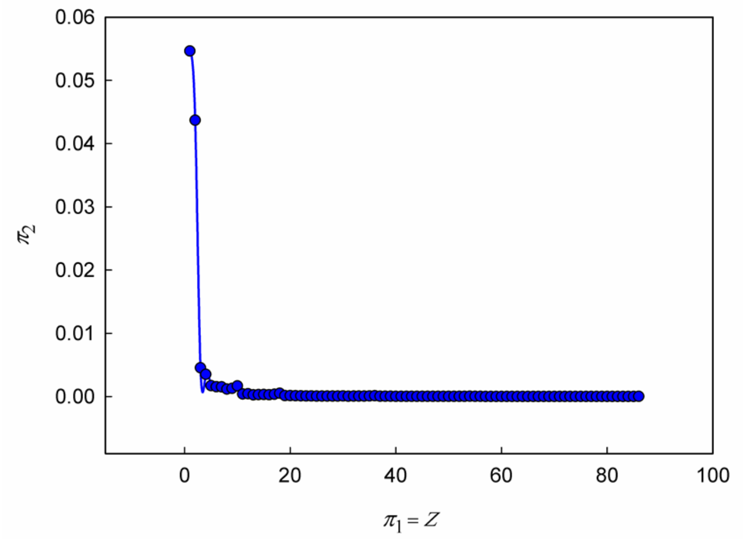

Fig. S2 Calculated dependence π_2 vs. π_1.

Formulae for approximate calculation of a_j and b_j values in the Fourier series (15):

$$a_j = \frac{2}{86}\sum_{k=0}^{85}\pi_i \cos j\frac{2\pi k}{85},$$

$$b_j = \frac{2}{86}\sum_{k=0}^{85}\pi_i \sin j\frac{2\pi k}{85},$$

Formulae for calculating the values of A_j and φ_j in the series (16):

$$A_j = \sqrt{a_j^2 + b_j^2},$$

$$\mathrm{tg}\varphi_j = \frac{b_j}{a_j}$$

Table S2 Fourier expansion parameters for the quantity π_2

j	a_j	b_j	A_j	φ_i
0	0.00281	0.00000	0.00281	0.00000
1	0.00267	0.00028	0.00268	0.10502
2	0.00253	0.00042	0.00256	0.16573
3	0.00240	0.00050	0.00245	0.20743
4	0.00230	0.00055	0.00236	0.23442
5	0.00223	0.00059	0.00231	0.26017
6	0.00215	0.00064	0.00225	0.28731
7	0.00209	0.00066	0.00220	0.30601
8	0.00205	0.00069	0.00217	0.32391
9	0.00202	0.00073	0.00215	0.34533
10	0.00199	0.00080	0.00214	0.38197
11	0.00191	0.00086	0.00210	0.42478
12	0.00182	0.00091	0.00204	0.46373
13	0.00173	0.00093	0.00197	0.49491
14	0.00165	0.00093	0.00190	0.51453
15	0.00159	0.00094	0.00185	0.53542
16	0.00152	0.00095	0.00179	0.55640
17	0.00146	0.00095	0.00174	0.57611
18	0.00140	0.00095	0.00170	0.59638
19	0.00135	0.00096	0.00166	0.61528
20	0.00129	0.00097	0.00162	0.64483
21	0.00122	0.00098	0.00156	0.67881
22	0.00113	0.00097	0.00149	0.70955
23	0.00105	0.00094	0.00141	0.73079
24	0.00100	0.00091	0.00135	0.73726
25	0.00095	0.00088	0.00129	0.74871
26	0.00090	0.00085	0.00124	0.75908
27	0.00085	0.00083	0.00118	0.77084
28	0.00080	0.00080	0.00113	0.78359
29	0.00076	0.00077	0.00108	0.79026
30	0.00071	0.00075	0.00103	0.81641
31	0.00064	0.00073	0.00097	0.84715
32	0.00057	0.00068	0.00089	0.87315
33	0.00051	0.00062	0.00080	0.88358
34	0.00047	0.00055	0.00072	0.86031
35	0.00044	0.00048	0.00066	0.83073
36	0.00042	0.00043	0.00060	0.79510
37	0.00039	0.00037	0.00054	0.75471
38	0.00037	0.00031	0.00048	0.69078
39	0.00035	0.00025	0.00043	0.61841
40	0.00033	0.00019	0.00038	0.53165
41	0.00030	0.00013	0.00033	0.39459
42	0.00028	0.00005	0.00029	0.16879

Table S3 Fourier expansion parameters for the quantity π_3

j	a_j	b_j	A_j	φ_i
0	0.12309	0.00000	0.12309	0.00000
1	0.00291	0.00079	0.00302	0.26514
2	0.00979	0.00600	0.01148	0.54931
3	0.00661	–0.00155	0.00679	–0.23015
4	–0.00528	–0.01079	0.01201	1.11594
5	0.02547	0.00025	0.02547	0.00965
6	–0.00162	–0.00447	0.00476	1.22356
7	0.00250	–0.00006	0.00250	–0.02507
8	0.00871	–0.00298	0.00921	–0.32950
9	0.00590	–0.01364	0.01486	–1.16224
10	0.01862	0.01023	0.02125	0.50220
11	0.01065	0.00235	0.01091	0.21752
12	0.00269	0.00432	0.00509	1.01441
13	0.00334	0.00672	0.00751	1.10916
14	0.00426	–0.00940	0.01033	–1.14503
15	0.00761	0.00529	0.00926	0.60729
16	0.00639	–0.00121	0.00651	–0.18645
17	0.00364	–0.00155	0.00396	–0.40272
18	0.00252	0.00236	0.00345	0.75127
19	0.00822	–0.00704	0.01083	–0.70808
20	0.00648	0.00521	0.00832	0.67710
21	0.00682	0.00512	0.00852	0.64364
22	0.00268	0.00268	0.00379	0.78638
23	–0.00485	0.00425	0.00645	–0.71951
24	0.00758	–0.00335	0.00829	–0.41652
25	0.00193	0.00135	0.00236	0.61115
26	0.00487	0.00302	0.00573	0.55430
27	0.00761	0.00058	0.00764	0.07652
28	–0.00318	–0.00032	0.00319	0.10027
29	0.01089	–0.00237	0.01115	–0.21446
30	0.00474	0.00195	0.00512	0.39055
31	0.00368	0.00530	0.00645	0.96416
32	0.00433	0.00418	0.00602	0.76738
33	–0.00724	–0.00042	0.00725	0.05813
34	0.00348	–0.00072	0.00356	–0.20390
35	0.00192	–0.00142	0.00238	–0.63645
36	0.00142	0.00083	0.00165	0.52872
37	0.00374	0.00533	0.00651	0.95836
38	0.00017	–0.00460	0.00461	–1.53489
39	0.00454	0.00294	0.00541	0.57451
40	0.00498	0.00281	0.00572	0.51430
41	0.00183	0.00187	0.00262	0.79514
42	–0.00082	0.00836	0.00840	–1.47304

Table S4 Fourier expansion parameters for the quantity π_4

j	a_j	b_j	A_j	φ_i
0	0.15369	0.00000	0.15369	0.00000
1	0.01586	0.01223	0.02003	0.65698
2	0.01511	0.00660	0.01649	0.41201
3	0.00476	0.00405	0.00626	0.70502
4	–0.00418	0.00516	0.00664	–0.89019
5	0.00600	–0.00025	0.00600	–0.04091
6	–0.00084	0.00466	0.00473	–1.39303
7	0.00090	0.00194	0.00214	1.13639
8	0.00484	–0.00155	0.00508	–0.31016
9	0.00221	–0.00776	0.00807	–1.29294
10	0.01774	–0.00261	0.01793	–0.14627
11	0.01173	0.00244	0.01199	0.20542
12	0.00934	0.00665	0.01147	0.61872
13	0.00817	0.00807	0.01149	0.77936
14	–0.00108	0.00042	0.00116	–0.36719
15	0.00743	0.00237	0.00780	0.30890
16	0.00287	0.00008	0.00287	0.02681
17	0.00292	0.00104	0.00310	0.34093
18	0.00449	0.00304	0.00542	0.59488
19	0.00384	–0.00185	0.00426	–0.44961
20	0.00846	0.00378	0.00927	0.42080
21	0.00630	0.00598	0.00868	0.75932
22	0.00244	0.00692	0.00734	1.23241
23	–0.00212	0.00723	0.00753	–1.28518
24	0.00240	0.00059	0.00247	0.24085
25	0.00109	0.00350	0.00366	1.26812
26	0.00332	0.00204	0.00389	0.55074
27	0.00507	0.00132	0.00524	0.25554
28	–0.00109	0.00218	0.00244	–1.10768
29	0.00757	–0.00153	0.00772	–0.19892
30	0.00476	0.00404	0.00625	0.70334
31	0.00326	0.00646	0.00724	1.10360
32	0.00194	0.00581	0.00613	1.24859
33	–0.00641	0.00525	0.00828	–0.68624
34	–0.00176	0.00059	0.00186	–0.32448
35	–0.00157	0.00083	0.00178	–0.48846
36	–0.00026	0.00130	0.00132	–1.37278
37	0.00025	0.00303	0.00304	1.48879
38	–0.00015	–0.00030	0.00034	1.11101
39	0.00143	0.00300	0.00333	1.12619
40	0.00118	0.00345	0.00365	1.24221
41	–0.00080	0.00250	0.00262	–1.26280
42	–0.00310	0.00540	0.00622	–1.04994

Table S5 Fourier expansion parameters for the quantity π_5

j	a_j	b_j	A_j	φ_i
0	1.78008	0.00000	1.78008	0.00000
1	–0.03631	–0.07118	0.07990	1.09908
2	0.00303	0.01606	0.01634	1.38425
3	0.03056	–0.01139	0.03261	–0.35672
4	–0.00015	–0.10112	0.10112	1.56930
5	0.15099	0.00470	0.15106	0.03111
6	–0.00115	–0.06059	0.06060	1.55185
7	0.01842	–0.02255	0.02911	–0.88591
8	0.04156	–0.02167	0.04688	–0.48067
9	0.06585	–0.05361	0.08491	–0.68332
10	0.03440	0.08549	0.09215	1.18825
11	0.04016	0.00874	0.04110	0.21421
12	–0.00748	–0.01072	0.01307	0.96120
13	–0.01067	0.00497	0.01177	–0.43574
14	0.06681	–0.03409	0.07500	–0.47189
15	0.00989	0.01922	0.02162	1.09535
16	0.03557	0.00243	0.03565	0.06807
17	0.03187	–0.01100	0.03371	–0.33251
18	0.01107	–0.01108	0.01566	–0.78595
19	0.04590	–0.00228	0.04596	–0.04973
20	0.00613	0.00904	0.01092	0.97542
21	0.01771	0.00494	0.01838	0.27189
22	0.01757	–0.01350	0.02216	–0.65510
23	0.00785	–0.02344	0.02472	–1.24749
24	0.03771	0.00351	0.03787	0.09289
25	0.02027	–0.01161	0.02336	–0.52015
26	0.01849	0.00370	0.01885	0.19755
27	0.03050	0.00310	0.03066	0.10131
28	0.02474	–0.01867	0.03100	–0.64645
29	0.02343	0.00611	0.02422	0.25506
30	0.02170	–0.00129	0.02174	–0.05928
31	0.02077	–0.00522	0.02142	–0.24614
32	0.02231	–0.00628	0.02318	–0.27428
33	0.02781	–0.01229	0.03040	–0.41607
34	0.02654	–0.00162	0.02659	–0.06095
35	0.02593	–0.00300	0.02610	–0.11511
36	0.02165	–0.00036	0.02165	–0.01677
37	0.02367	0.00228	0.02378	0.09616
38	0.03017	–0.00596	0.03075	–0.19502
39	0.02044	–0.00326	0.02069	–0.15797
40	0.02378	0.00202	0.02386	0.08482
41	0.02862	–0.00042	0.02862	–0.01456
42	0.02614	–0.00063	0.02615	–0.02392

The main results of alternative calculations when using the values of atomic radii borrowed from Clementi et al. [51]:

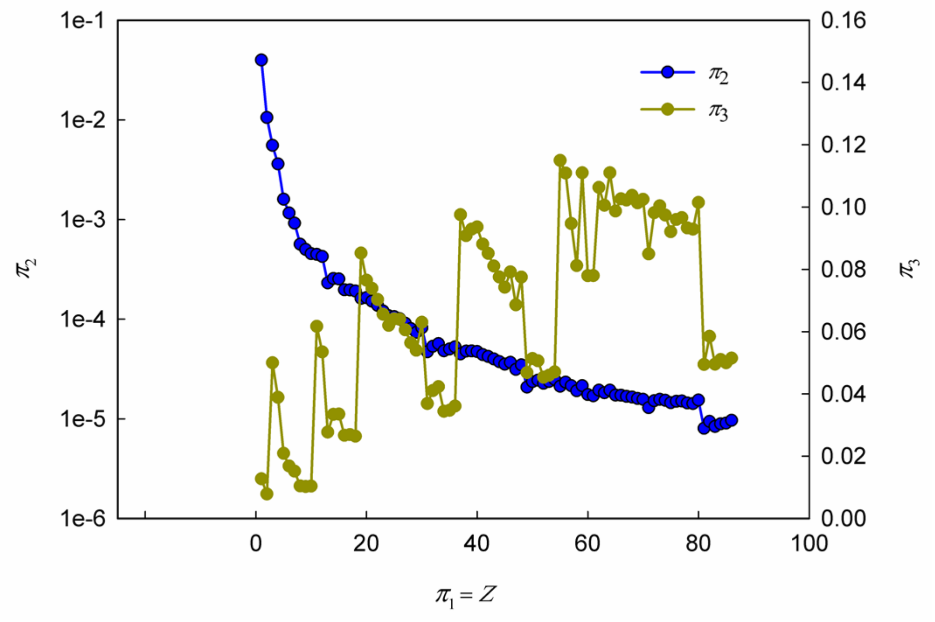

Fig. S3 Calculated dependences π_2 vs. π_1 and π_3 vs. π_1. For convenience, the values on the π_2-axis are plotted in logarithmic coordinates.

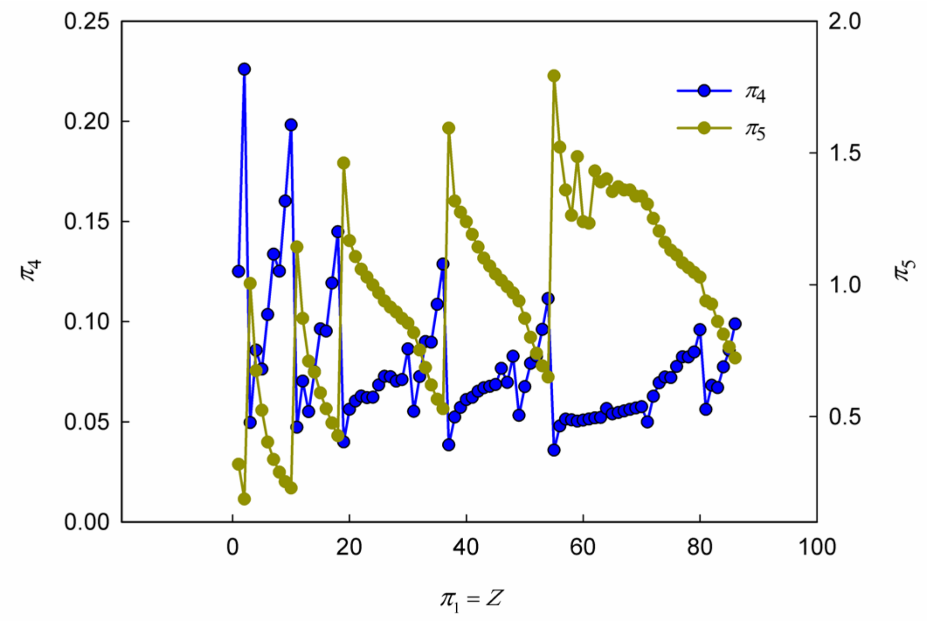

Fig. S4 Calculated dependences π_4 vs. π_1 and π_5 vs. π_1.

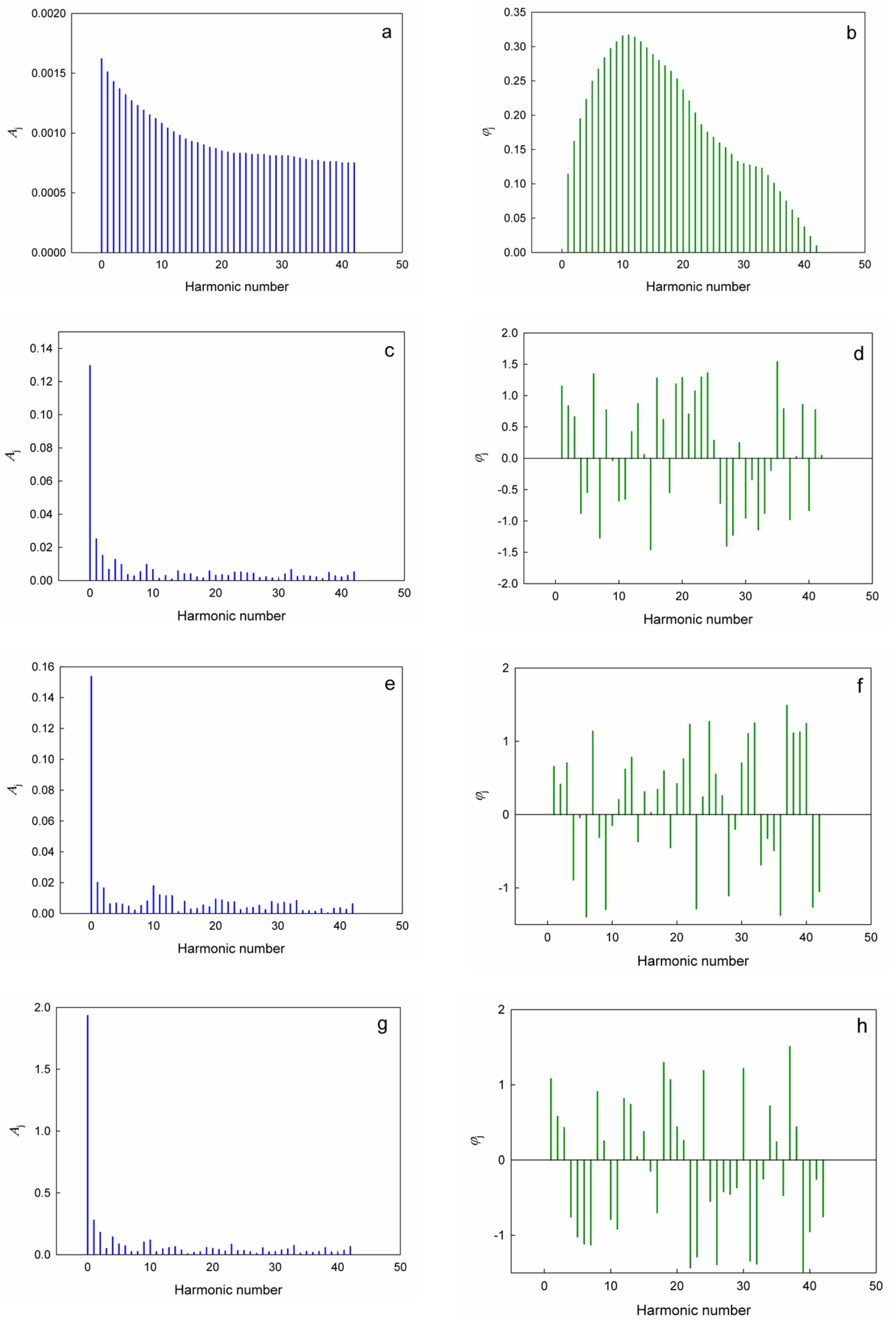

Fig. S5 Amplitude spectra (**a**, **c**, **e**, and **g**) and frequency spectra (**b**, **d**, **f**, and **h**) of the expansions of the dimensionless complex quantities π_2 (**a** and **b**), π_3 (**c** and **d**), π_4 (**e** and **f**) and π_5 (**g** and **h**) in the Fourier series (16).
